# Aspirin-Mediated Attenuation of Intervertebral Disc Degeneration by Ameliorating Reactive Oxygen Species *In Vivo* and *In Vitro*

**DOI:** 10.1155/2019/7189854

**Published:** 2019-11-06

**Authors:** Yu Liu, Jiayi Lin, Xiexing Wu, Xiaobin Guo, Houyi Sun, Binqing Yu, Jining Shen, Jiaxiang Bai, Zhanghuan Chen, Huilin Yang, Dechun Geng, Haiqing Mao

**Affiliations:** Department of Orthopaedics, The First Affiliated Hospital of Soochow University, Suzhou 215006, China

## Abstract

Intervertebral disc (IVD) degeneration (IDD) is a major cause of low back pain. The pathogenesis of IDD is associated with the disturbance of reactive oxygen species (ROS) equilibrium, inflammation, and matrix loss. Aspirin is a nonsteroidal anti-inflammatory drug that effectively inhibits inflammation and oxidative stress and has been widely used for the treatment of back pain. Therefore, we hypothesize that aspirin reverses the IDD process via antioxidative and anti-inflammatory effects on the AMPK signaling pathway. *In vitro*, aspirin diminished cellular oxygen free radicals (ROS, nitric oxide (NO)) and inflammatory cytokines (interleukin- (IL-) 1*β* and IL-6 and tumor necrosis factor alpha (TNF-*α*)) induced by lipopolysaccharides (LPS) in nucleus pulposus cells (NPCs). We found that aspirin preserved the extracellular matrix (ECM) content of collagen type II (COL2) and aggrecan while inhibiting the expression of matrix-degenerating enzymes, including matrix metalloproteinase 3 and 13 (MMP-3 and MMP-13) and A disintegrin and metalloproteinase with thrombospondin motifs 4 and 5 (ADAMTS-4, ADAMTS-5). Aspirin significantly promoted the ratios of p-AMPK to AMPK and p-ACC to ACC expression in NPCs. Furthermore, pretreatment with the AMPK inhibitor compound C abrogated the antioxidant effects of aspirin. *In vivo*, an IDD model was established in Sprague-Dawley rats via percutaneous disc puncture with the 20-gauge needle on levels 8-9 and 9-10 of the coccygeal vertebrae. Imaging assessment showed that after aspirin treatment, improvements in disc height index (DHI) ranged from 1.22-fold to 1.54-fold and nucleus pulposus signal strength improved from 1.26-fold to 1.33-fold. Histological analysis showed that aspirin treatment prevented the loss of COL2 and decreased MMP-3 and MMP-13, inducible NO synthase (iNOS), cyclooxygenase-2 (COX-2), IL-1*β*, and TNF-*α* expression in the IVD tissues. These results suggest that treatment with aspirin could reverse the IDD process via the AMPK signaling pathway, which provides new insights into the potential clinical applications of aspirin, particularly for IDD treatment.

## 1. Introduction

In the past few decades, the prevalence of degenerative disc diseases has increased, and low back pain is now a major health problem [1, 2]. Researchers have suggested that more than half of musculoskeletal disability cases are caused by degenerative disc diseases. The United States spends more than $100 billion every year for the treatment of degenerative disc diseases [3]. Currently, relevant clinical therapy for degenerative disc diseases mainly includes surgery and conservative treatment, which are aimed at achieving symptomatic relief rather than preventing intervertebral disc (IVD) degeneration (IDD) [4].

The pathogenesis of IDD remains unclear, but it is typically characterized by matrix dehydration in the nucleus pulposus, loss of disc height, and mechanical dysfunction [5–7], thereby resulting in traumatic injury and pain. Some investigators have found that multiple pathological changes can enhance the levels of inflammatory cytokines, such as interleukin-1*β* (IL-1*β*) and tumor necrosis factor alpha (TNF-*α*). These cytokines can increase the expression of A disintegrin and metalloproteinase with thrombospondin motifs 4 and 5 (ADAMTS-4, ADAMTS-5) and matrix metalloproteinase 3 and 13 (MMP-3, MMP-13) and promote aggrecan degradation [8, 9]. Associations among herniation, IDD, and the inflammatory response have been well established [9]. The IDD tissue contains many inflammatory cells which secrete TNF-*α*, IL-1*β*, and other cytokines found in the tissue at the injury site [10]. Furthermore, these inflammatory cytokines are upregulated with age and IDD progression and are positively correlated with the degree of pain [11]. Importantly, the expression of inflammatory cytokines is not only associated with degeneration but is also considered as its cause.

Recent research has suggested that oxidative stress plays an important role in the development of degeneration [12–14]. During oxidative stress, excessive amounts of reactive oxygen species (ROS), such as superoxide anions, hydroxyl radicals, and hydrogen peroxide, are generated by the mitochondria [15]. Excessive ROS levels can directly impair nucleus pulposus cells (NPCs), interfere with the homeostasis of the IVD matrix, reduce the synthesis of proteoglycans, and increase the expression of MMPs [16, 17]. ROS and chronic inflammation are interdependent processes that have been correlated with degeneration [18]. Research indicates that ROS can promote the expression of inflammatory cytokines in the degenerated tissue [19]. Therefore, decreasing the expression of ROS could alleviate IDD.

Aspirin is a salicylate that was introduced in the late 1890s and has been used for the treatment of a variety of inflammatory diseases [20]. Traditionally, aspirin has been utilized to reduce the risk of cardiovascular disease and in the treatment of rheumatism or arthritis [21]. It has been recently reported in several studies that a low-dose aspirin administration can prevent preeclampsia [22], inhibit platelets to increase T cell activity [23], and attenuate ROS expression in microglia [24, 25]. It has also been suggested that aspirin could reduce oxidative stress and inflammation, which in turn may reverse the IDD process.

The present study induced oxidative stress and inflammation in NPCs using lipopolysaccharides (LPS) to assess the capability of aspirin to improve IDD based on its antioxidant activity and possible relevant signaling pathways. Our results clearly show that aspirin could significantly improve IDD via its antioxidative and anti-inflammatory effects *in vivo* and *in vitro*. Importantly, our data indicated that aspirin exerts its protective effects through the 5′ adenosine monophosphate-activated protein kinase (AMPK) signaling pathway. These results improve our understanding of the underlying mechanism by which aspirin inhibits IDD.

## 2. Materials and Methods

### 2.1. Ethics Statement

All of the experiments performed in this study were approved by the Ethics Committee of the First Affiliated Hospital of Soochow University (Suzhou, China) (No. 201801A005). All of the animal surgical interventions and treatments and animal care were conducted in strict accordance with the directives of the Laboratory Animal Center of Soochow University, Suzhou, China.

### 2.2. Experimental Animals

We purchased Sprague-Dawley (SD) rats at osseous maturity (male, age: 3 months, weight: 450 g ± 50 g) from the Laboratory Animal Center of Soochow University. The animals were kept in a ventilated environment with a 12 h/12 h light-dark cycle at a constant temperature of 21°C.

### 2.3. Reagents and Antibodies

We purchased aspirin (V900185), LPS (*Escherichia coli* 0111:B4), and phosphate-buffered saline (PBS) from Sigma-Aldrich (St. Louis, Missouri, USA). A 2′,7′-dichlorodihydrofluorescein diacetate (DCFH-DA) fluorescent probe (Beyotime Institute of Biotechnology, Shanghai, China) was used to assess intracellular ROS production, and 5-aminoimidazole-4-carboxamide ribonucleotide (AICAR) and compound C were purchased from Selleck Chemicals (Shanghai, China). Additionally, we purchased rabbit anti-rat collagen type II (COL2; ab34712), MMP-3 (ab13533), MMP-13 (ab39012), IL-1*β* (ab9722), TNF-*α* (ab6671), AMPK (ab32047), phospho-AMPK (p-AMPK; ab133448), acetyl-CoA carboxylase (ACC; ab45174), phospho-ACC (p-ACC, ab68191), nitric oxide (NO) synthase (iNOS; ab15323), cyclooxygenase-2 (COX-2; ab15191), and nuclear factor (erythroid-derived 2)-like 2 (Nrf-2; ab137550) antibodies, as well as goat anti-rabbit immunoglobulin G (IgG) heavy+light chain (H&L) Alexa Fluor 488 (ab150077) and Alexa Fluor 647 (ab150079) from Abcam (Cambridge, UK). Anti-*β*-actin and 4′,6-diamidino-2-phenylindole (DAPI) were purchased from Beyotime. Aggrecan (bs-1223R), ADAMTS-4 (bs-4191R), and ADAMTS-5 (bs-3573R) were purchased from Bioss (Beijing, China).

### 2.4. NPC Culture

NPCs derived from normal human individuals are difficult to obtain and are usually obtained from teenagers who developed vertebral body fracture bursts. Different types and degrees of IDD largely influence the traits of the obtained NPCs. For these reasons, in this study, we used Sprague-Dawley (SD) rat NPCs. SD rats were given an overdose of sodium pentobarbital (100 mg/kg) and then sacrificed. The nucleus pulposus was carefully separated from the annular fibrous tissues and collected from the caudal discs (Co1–Co5) under aseptic conditions and a Stemi 305 dissecting microscope (Zeiss, Oberkochen, Germany). The tissues were dissociated using 0.5% (*w*/*v*) collagenase type II for 2 h at 37°C. Next, the digested tissues were cultured in Dulbecco's modified Eagle's medium/Nutrient Mixture F-12 (DMEM/F-12; Invitrogen, Waltham, MA, USA) supplemented with 15% fetal bovine serum (FBS; Invitrogen) and antibiotics (1% penicillin and streptomycin) in an incubator maintained at 5% carbon dioxide and at 37°C. Upon reaching confluency, the cells were harvested using 0.25% trypsin-ethylenediaminetetraacetic acid (EDTA; Invitrogen). The medium was changed every 3 d. The NPCs were subsequently divided into five experimental groups. (1) Control group: the NPCs were treated with DMEM/F-12 for 24 h. (2) LPS group: the NPCs were cultured in DMEM/F-12 with LPS (1 *μ*g/mL) for 24 h. (3) Aspirin+LPS group: the NPCs were pretreated with DMEM/F-12 supplemented with aspirin (5 or 25 *μ*g/mL) for 3 h and then cultured in DMEM/F-12 with LPS (1 *μ*g/mL) and aspirin (5 or 25 *μ*g/mL) for 24 h. (4) Aspirin+LPS+compound C (AMPK inhibitor) group: the NPCs were pretreated with compound C (100 *μ*M) for 24 h, then with DMEM/F-12 supplemented with aspirin (25 *μ*g/mL) for 3 h, and finally with DMEM/F-12 with LPS (1 *μ*g/mL) and aspirin (25 *μ*g/mL) for 24 h. (5) LPS+AICAR (AMPK agonist) group: the NPCs were pretreated with AICAR (500 *μ*M) for 24 h, then cultured with DMEM/F-12 supplemented with LPS (1 *μ*g/mL) for 24 h.

### 2.5. Cell Viability Assay

To assess the effects of different reagents, we seeded NPCs (density: 3 × 10^3^ cells per well) in a 96-well plate and viability was examined using a Cell Counting Kit-8 (CCK-8; Dojindo Co., Kumamoto, Japan) following the manufacturer's protocols. The NPCs were treated with different concentrations of aspirin and LPS for different time periods. Optical density (OD) was measured at a wavelength of 450 nm using a Gen5 microplate reader (BioTek Instruments, Inc., Winooski, VT, USA).

### 2.6. Measurement of ROS Production

NPCs (density: 1 × 10^5^ cells per well) in a 24-well plate were pretreated with different concentrations of aspirin and incubated with 1 *μ*g/mL LPS. Subsequently, these were incubated with 10 *μ*M DCFH-DA for 30 min in the dark. The cells were then assessed in terms of ROS-mediated fluorescence under an inverted fluorescence microscope (IFM) and by flow cytometry (FCM) using fluorescein isothiocyanate (FITC) [26]. Under the FITC fluorescence channel in IFM, typical positive cells were labeled as bright green. The average fluorescence intensity can be calculated by counting by FCM in the FITC channel and for comparison.

### 2.7. Assessment of NO Production

We measured NO using a nitrite detection kit (Beyotime Institute of Biotechnology, Jiangsu, China). Briefly, we collected a 50 *μ*L sample of the cell culture medium and mixed this with 50 *μ*L each of Griess Reagents I and II from the kit. We then measured the absorbance of the mixture at a wavelength of 540 nm and calculated nitrite concentration using a standard curve of sodium nitrite [27].

### 2.8. Enzyme-Linked Immunosorbent Assay (ELISA)

We determined IL-1*β*, IL-6, and TNF-*α* levels in the cell culture supernatants using an ELISA kit (Hangzhou MultiSciences Biotech Co. Ltd., Hangzhou, China). Briefly, the NPCs were cultured to confluency in 24-well plates (density: 5 × 10^4^ cells per well) and pretreated with or without aspirin, followed by incubation with LPS (1 *μ*g/mL). At the end of the treatment period, we transferred 100 *μ*L aliquots of cell culture media to wells coated with anti-IL-1, anti-IL-6, or anti-TNF-*α* antibody for 2 h. Then, the wells were thoroughly washed, followed by the addition of 50 *μ*L of the E-selectin antibody to each well and incubation for 45 min to allow coating of the well. After incubation, the wells were immediately washed, and a peroxidase-conjugated secondary polyclonal antibody solution was added to each well and incubated for 30 min. After washing to remove any unbound antibodies, we added a substrate solution to each well and allowed the reaction to develop for 20 min. Color development was then stopped, and absorbance was measured at a wavelength of 450 nm using a microplate reader.

### 2.9. Reverse Transcription Quantitative Polymerase Chain Reaction (RT-qPCR)

We extracted total ribonucleic acid (RNA) using TRIzol reagent (Invitrogen), and then total RNA concentrations were determined using a NanoDrop 2000 (Thermo Fisher Scientific, Waltham, MA, USA). For reverse transcription, the PrimeScript RT Master Mix kit (Takara, Kusatsu, Japan) was used, and 1 *μ*g of reverse transcription product (cDNA) was used for PCR amplification. PCR amplification required 20 *μ*L of the reverse transcription product in each reaction, plus 10 *μ*L Forget-Me-Not qPCR Master Mix (Biotium, Inc., Hayward, CA, USA), 0.5 *μ*L of each primer, 1 *μ*L of cDNA, and 8 *μ*L RNase-free distilled water (dH_2_O; Invitrogen). The reactions were performed using the CFX96 Touch Real-Time PCR Detection System (Bio-Rad Laboratories, Hercules, CA, USA). Cycle threshold values were normalized to the level of glyceraldehyde 3-phosphate dehydrogenase (GAPDH). To calculate messenger RNA levels, we used the 2^−*ΔΔ*CT^ method [28]. The primers sequences are presented in Supplemental [Supplementary-material supplementary-material-1].

### 2.10. Western Blot Assay

Total protein extraction was performed in a radioimmunoprecipitation assay (RIPA) supplemented with phenylmethanesulfonyl fluoride (PMSF), and protein concentration was measured with a bicinchoninic acid (BCA) kit (Beyotime, Shanghai). Approximately 30 *μ*g of protein was separated via sodium dodecyl sulfate polyacrylamide gel electrophoresis (SDS-PAGE) and transferred onto polyvinylidene difluoride (PVDF) membranes (Bio-Rad). After blocking with 5% nonfat milk, the membranes were incubated with primary antibodies against *β*-actin (1 : 5,000), AMPK (1 : 500), p-AMPK (1 : 500), ACC (1 : 1,000), p-ACC (1 : 1,000), iNOS (1 : 250), COX-2 (1 : 1,000), and Nrf-2 (1 : 1,000) overnight at 4°C, followed by the respective secondary antibodies. Finally, we detected bands using the Electrochemiluminescence Plus Reagent (Thermo Fisher) and quantified their intensity with Image Lab software version 3.0 (Bio-Rad).

### 2.11. Immunocytochemical (ICC) Staining

Cells were cultured on 24-well plates. After washing with PBS, the cells were fixed with formaldehyde for 30 min and incubated in 2% fetal calf serum for 2 h. Then, the cells were incubated overnight with COL2 (1 : 200), aggrecan (1 : 200), MMP-3 (1 : 200), MMP-13 (1 : 200), ADAMTS-4 (1 : 150), and ADAMTS-5 (1 : 150) rabbit anti-rat antibodies and then with goat anti-rabbit IgG H&L Alexa Fluor 488 or 647 (1 : 1,000) for 2 h, followed by counterstaining with DAPI for 30 min.

### 2.12. Surgical Procedures

After 12 h of fasting and 4 h of water deprivation, we anesthetized the animals via intraperitoneal injection of 0.3% pentobarbital. An IDD model was established as described by Han et al.'s [29] method and by adopting a standard surgical procedure. The areas between the eighth and ninth coccygeal vertebrae (Co8–Co9) and between the ninth and tenth vertebrae (Co9–Co10) were punctured using a 20-gauge needle. To ensure degeneration, we punctured the annulus fibrosus and then rotated the needle for 5 s and held it steady for 30 s. To avoid possible influences of individual differences, the following groups were established: (1) control group: nonpunctured Co7–Co8 discs; (2) aspirin group: punctured Co8–Co9 discs and injected aspirin; and (3) IDD group: punctured Co9–Co10 discs and injected PBS. Additionally, we randomly divided the aspirin-injected rats into subgroups that received low (10 *μ*g/mL; *n* = 20) and high (100 *μ*g/mL; *n* = 20) concentrations of aspirin. Similar to our previous research, we eliminated the influences of injected volume by injecting only 2 *μ*L of PBS or high- or low-concentration aspirin into the center space of the nucleus pulposus at a depth of 6 mm [30, 31]. To avoid secondary damage caused by puncture, all of the injections were performed with a 33-gauge Hamilton syringe (Hamilton Co., Reno, NV, USA) 3 days after the day of puncture.

### 2.13. Histological Analysis

We fixed the rat caudal discs with 4% paraformaldehyde for 48 h and decalcified these in 10% EDTA for 45 days. The fixed Co7–Co10 samples then underwent paraffin embedding. Sections (5 *μ*m in thickness) were cut and then stained with either hematoxylin and eosin (H&E) or Safranin O Fast Green. We determined the histological grade according to Masuda [32].

### 2.14. Immunohistochemistry (IHC) Staining

After dewaxing, gradient dehydration, and antigen retrieval, we selected representative indicators of inflammation (IL-1*β* (1 : 100), TNF-*α* (1 : 150)), oxidative stress (iNOS (1 : 100), COX-2 (1 : 100)), and degeneration (COL2 (1 : 200) and MMP-3 (1 : 150)), added their respective primary antibodies to the sections, and incubated these in the dark at 4°C for 12 h. Then, the sections were incubated at room temperature in a buffer with secondary antibodies for 35 min. After incubation, the sections were rinsed and then counterstained with hematoxylin. We quantified each group using Image Pro-Plus image analysis software version 6.0 (Media Cybernetics, Rockville, MD, USA).

### 2.15. X-Ray and Magnetic Resonance Imaging (MRI) Analysis

Seven days after the initial annulus puncture, each group of rats (*n* = 20) was randomly selected to undergo X-ray (*n* = 10) and MRI (*n* = 10) scans before these were sacrificed. The rats were kept in a supine position with their tails straight for placement on a molybdenum target radiographic-image unit (General Electric, Boston, MA, USA). Radiographs were taken at a collimator-to-film distance of 66 cm, an exposure of 63 mAs, and a penetration power of 35 kV. We performed MRI using a 1.5 T system (GE) to obtain T2-weighted images (repletion time: 3,000 ms; echo time: 80 ms; field of view: 200 mm^2^; slice thickness: 1.4 mm) on the coronal plane. All of the radiographic images were saved in a Neusoft PACS/RIS DICOM 3.0 medical imaging system (Neusoft, Liaoning, China). IVD height and the adjacent upper and lower vertebral-body heights were measured using Neusoft PACS/RIS measuring tools; from these values, the disc height index (DHI) was calculated.

### 2.16. Statistical Analysis

We used SPSS software version 25 (SSPS, Inc., Chicago, IL, USA) for statistical analysis. Data were expressed in terms of fold change, and experiments were conducted independently at least thrice. Statistical significance was determined by using one-way ANOVA and Tukey's honest significant-difference (HSD) tests. Differences in data were further analyzed between each group; mean differences were expressed with 95% confidence intervals (CIs). Differences were considered statistically significant at *P* < 0.05.

## 3. Results

### 3.1. Aspirin Attenuates Oxidative Stress and Expression of Proinflammatory Cytokines in NPCs

LPS stimulated ROS expression in a dose- and time-dependent manner without toxic effects (Supp. Figs. [Supplementary-material supplementary-material-1]), significantly upregulated MMP-3, and inhibited COL2 in NPCs (Supp. Fig. [Supplementary-material supplementary-material-1]). LPS also upregulated iNOS and COX-2 and downregulated Nrf-2 (Supp. Figs. [Supplementary-material supplementary-material-1]). These results were concordant to the findings of previous studies [33, 34].

**Figure 1:**
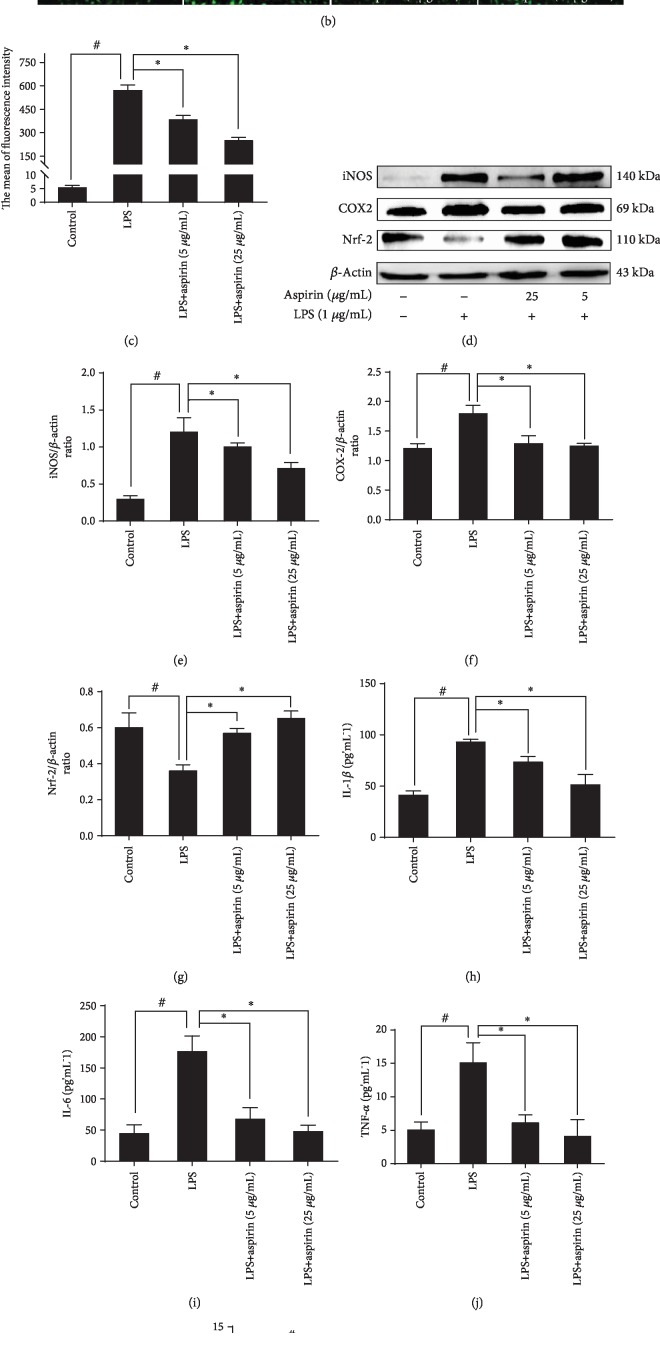
Aspirin attenuates oxidative stress and inflammation in NPCs. (a) NPCs were cultured with different concentrations of aspirin (0, 5, 25, 50, 75, and 100 *μ*g/mL) for 1, 3, and 5 d; cell viability was detected by CCK-8 (^∗^*P* < 0.05 vs. control group). NPCs were pretreated with aspirin (5 or 25 *μ*g/mL) for 3 h and then stimulated by 1 *μ*g/mL LPS for 24 h. (b) Fluorescence images show ROS levels in different groups. Scale bar, 400 *μ*m. (c) Average fluorescence intensity of ROS detected by FCM (^#^*P* < 0.05 vs. control group; ^∗^*P* < 0.05 vs. LPS group). (d–g) Western blotting results of the iNOS, COX-2, and Nrf-2 (^#^*P* < 0.05 vs. control group; ^∗^*P* < 0.05*vs.* LPS group). (h–j) ELISA-detected inflammatory cytokines IL-1*β*, IL-6, and TNF-*α*. (k) NO levels in different groups (^#^*P* < 0.05*vs*. control group; ^∗^*P* < 0.05*vs*. LPS group). ^#^*P* and ^∗^*P* < 0.05 by one-way ANOVA and Tukey's HSD test were further analyzed between LPS group *vs*. control group, aspirin (5 *μ*g/mL) group *vs*. LPS group, and aspirin (25 *μ*g/mL) group *vs.* LPS group (*n* = 3 independent experiments).

Figure 1(a) shows that aspirin (<100 *μ*g/mL) is not toxic to NPCs. Both aspirin dosages significantly mitigated ROS expression within the range of 32.9% (5 *μ*g/mL) to 56.4% (25 *μ*g/mL) compared to the LPS group (*P* < 0.05; Figures 1(b) and 1(c)). Western blotting showed that aspirin significantly decreased protein expression of iNOS (*P* < 0.05) and COX-2 (*P* < 0.05). In addition, aspirin treatment increased the expression of Nrf-2 (*P* < 0.05), which is a key protein involved in antioxidant responses (Figures 1(d)–1(g)).

In response to oxidative stress, NPCs secrete a series of inflammatory cytokines. Our results indicated that IL-1*β*, IL-6, and TNF-*α* expression markedly increased following LPS induction. Specifically, IL-1*β* expression increased by ~2.3-fold (Figure 1(h)), IL-6 increased by ~3.9-fold (Figure 1(i)), and TNF-*α* increased by ~2.8-fold (*P* < 0.05) (Figure 1(j)). Treatment with aspirin reversed the elevated expression of these inflammatory cytokines (Figures 1(h)–1(j)). We also evaluated the extracellular concentration of NO, which increased ~12.2-fold (*P* < 0.05) after LPS intervention. Aspirin reversed this decrease (Figure 1(k)).

### 3.2. Aspirin Alleviates LPS-Induced NPC Dysfunction

**Figure 2:**
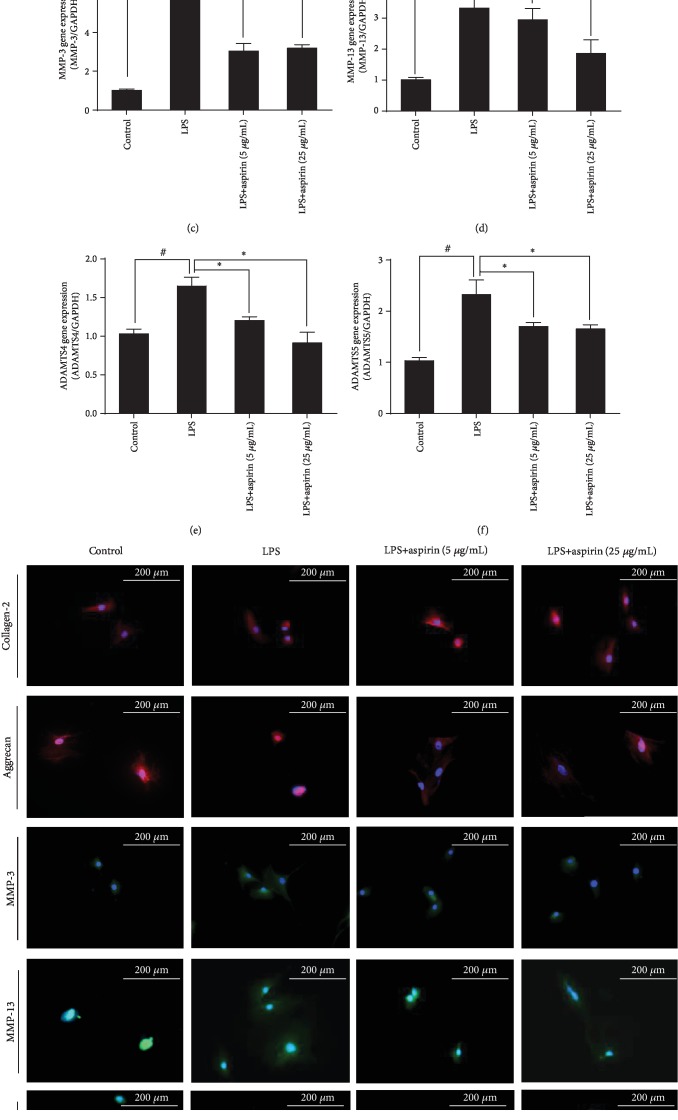
Aspirin alleviates LPS-induced degeneration of NPCs. NPCs were pretreated with aspirin (5 or 25 *μ*g/mL) for 3 h, then stimulated by 1 *μ*g/mL LPS for 24 h. (a–f) RT-qPCR results for COL2, aggrecan, MMP-3, MMP-13, ADAMTS-4, and ADAMTS-5 (^#^*P* < 0.05 vs. control group; ^∗^*P* < 0.05*vs.* LPS group). (g) Representative ICC image with COL2, aggrecan, MMP-3, MMP-13, ADAMTS-4, and ADAMTS-5 staining in NPCs. Scale bar, 200 *μ*m. ^#^*P* and ^∗^*P* < 0.05 by one-way ANOVA and Tukey's HSD test were further analyzed between LPS group *vs*. control group, aspirin (5 *μ*g/mL) group *vs*. LPS group, and aspirin (25 *μ*g/mL) group *vs*. LPS group (*n* = 3 independent experiments).

RT-PCR analysis indicated that LPS reduced the expression of COL2 and aggrecan (*P* < 0.05; Figures 2(a) and 2(b)), whereas it increased that of MMP-3 and MMP-13 (*P* < 0.05; Figures 2(c) and 2(d)) and the upstream regulatory factors ADAMTS-4 and ADAMTS-5 of MMPs (*P* < 0.05; Figures 2(e) and 2(f)). Aspirin reversed these changes (Figures 2(a)–2(f)). ICC staining showed that COL2 had a greater distribution around the nucleus in NPCs than in the LPS group and is full of aggrecan. In addition, the expression and distribution of MMP-3, MMP-13, ADAMTS-4, and ADAMTS-5 decreased after treatment with aspirin compared to the LPS group (Figure 2(g)).

### 3.3. Aspirin Plays a Protective Role via the AMPK Signaling Pathway

**Figure 3:**
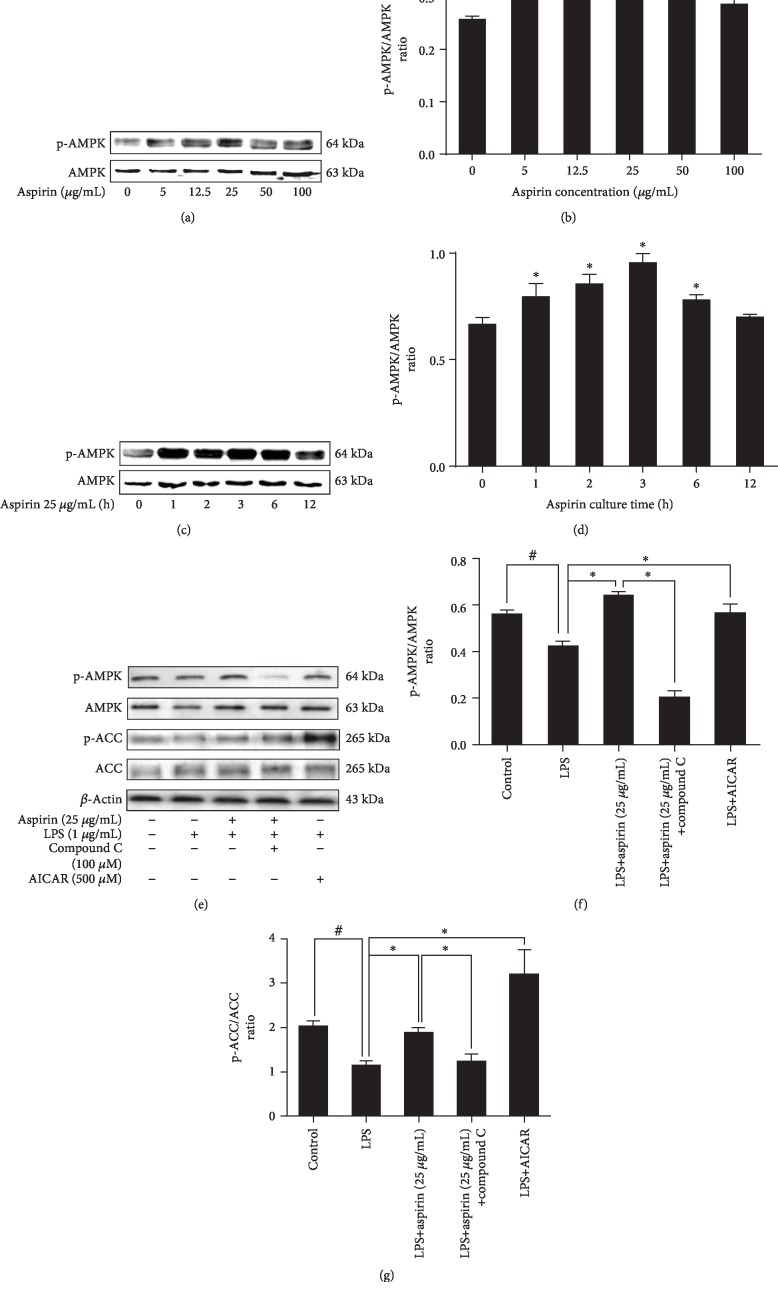
Aspirin activates the AMPK signaling pathway. NPCs were treated with different concentrations of aspirin (0, 5, 12.5, 25, 50, and 100 *μ*g/mL) for 3 h. (a, b) Western blotting results for the p-AMPK/AMPK ratio (^∗^*P* < 0.05*vs*. control group). NPCs were treated with aspirin (25 *μ*g/mL) for different time periods (0, 1, 2, 3, 6, 12, and 24 h). (c, d) Western blotting results for the p-AMPK/AMPK ratio (^∗^*P* < 0.05*vs.* control group). NPCs were pretreated with AMPK inhibitors (compound C, 100 *μ*M) or AMPK agonists (AICAR, 500 *μ*M) for 24 h; aspirin (25 *μ*g/mL) or LPS (1 *μ*g/mL) was then added for 3 h. (e–g) Western blot results for the p-AMPK/AMPK and p-ACC/ACC ratios. ^#^*P* and ^∗^*P* < 0.05 by one-way ANOVA and Tukey's HSD test were further analyzed between LPS group *vs*. control group, aspirin (25 *μ*g/mL) group *vs.* LPS group, aspirin (25 *μ*g/mL)+LPS+compound C group *vs.* aspirin (25 *μ*g/mL) group, and LPS+AICAR group *vs*. LPS group (*n* = 3 independent experiments).

The AMPK signaling pathway is closely related to oxidative stress. We hypothesized that aspirin achieves its antioxidative stress effect by activating this pathway. Our results indicated that aspirin-induced AMPK phosphorylation is dose- (Figures 3(a) and 3(b)) and time-dependent (Figures 3(c) and 3(d)). In addition, when the aspirin dosage was 25 *μ*g/mL, the ratio of p-AMPK to AMPK sharply increased, peaking at 3 h (*P* < 0.05; Figures 3(c) and 3(d)). To further explore whether aspirin could activate AMPK, we detected the protein ACC, which is downstream of AMPK. Our data showed that treatment with aspirin markedly increased ACC phosphorylation compared to the LPS group (*P* < 0.05; Figures 3(e)–3(g)).

**Figure 4:**
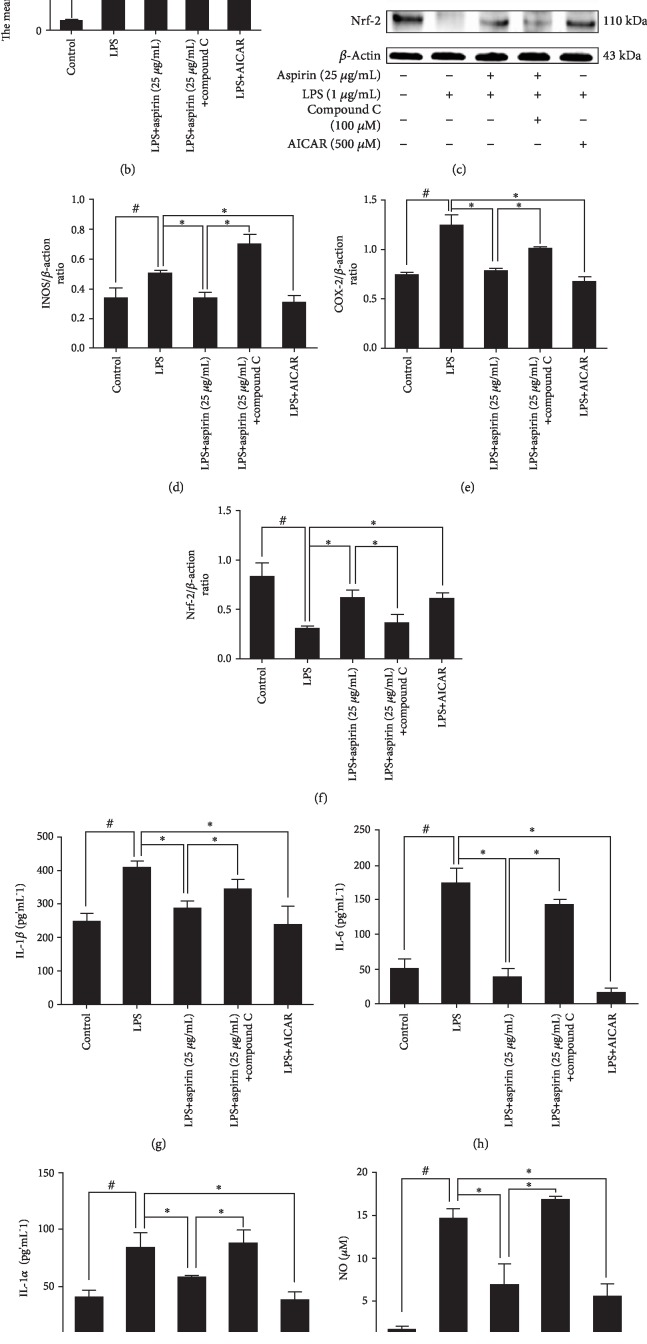
Activation of the APMK signaling pathway is essential to the antioxidative and anti-inflammatory effects of aspirin. NPCs were pretreated with an AMPK inhibitor (compound C, 100 *μ*M) or an AMPK agonist (AICAR, 500 *μ*M) for 24 h; aspirin (25 *μ*g/mL) or LPS (1 *μ*g/mL) was then added for 24 h. (a) Fluorescence images show ROS levels in different groups. Scale bar, 400 *μ*m. (b) Average fluorescence intensity of ROS detected by FCM (^#^*P* < 0.05*vs*. control group; ^∗^*P* < 0.05*vs.* LPS group or aspirin (25 *μ*g/mL) group). (c) Western blotting results for (d–f) iNOS, COX-2, and Nrf-2 (^#^*P* < 0.05*vs.* control group; ^∗^*P* < 0.05*vs*. LPS group or aspirin (25 *μ*g/mL) group). (g–i) ELISA-detected inflammatory cytokines IL-1*β*, IL-6, and TNF-*α*. (j) NO levels in different groups and ^#^*P* and ^∗^*P* < 0.05 by one-way ANOVA and Tukey's HSD test were further analyzed between LPS group *vs*. control group, aspirin (25 *μ*g/mL) group *vs.* LPS group, aspirin (25 *μ*g/mL)+LPS+compound C group *vs.* aspirin (25 *μ*g/mL) group, and LPS+AICAR group *vs.* LPS group (*n* = 3 independent experiments).

**Figure 5:**
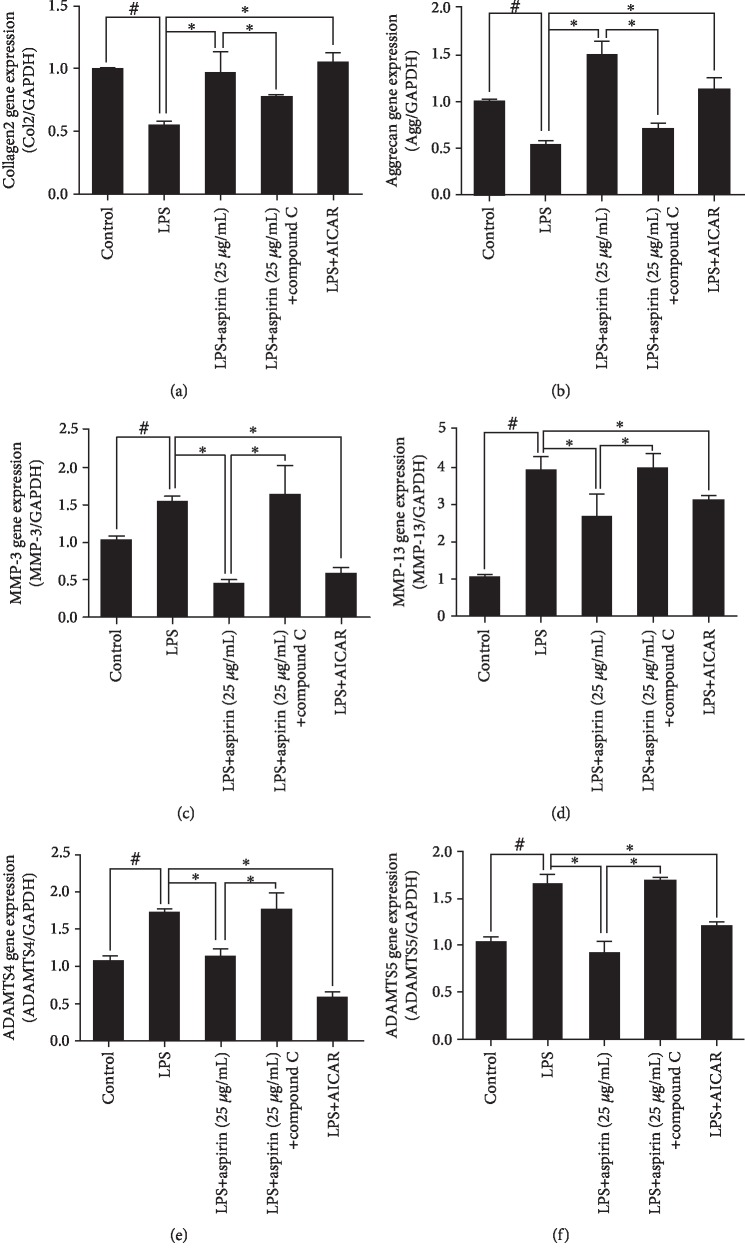
Inhibition of the APMK signaling pathway reverses the protective effects of aspirin on NPC dysfunction. NPCs were pretreated with an AMPK inhibitor (compound C, 100 *μ*M) or an AMPK agonist (AICAR, 500 *μ*M) for 24 h; aspirin (25 *μ*g/mL) or LPS (1 *μ*g/mL) was then added for 24 h. Subsequently, we obtained RT-PCR results for (a–f) COL2, aggrecan, MMP-3, MMP-13, ADAMTS-4, and ADAMTS-5 (^#^*P* < 0.05*vs.* control group; ^∗^*P* < 0.05*vs.* LPS group or aspirin (25 *μ*g/mL) group). LPS group *vs*. control group, aspirin (25 *μ*g/mL) group *vs.* LPS group, aspirin (25 *μ*g/mL)+LPS+compound C group *vs.* aspirin (25 *μ*g/mL) group, and LPS+AICAR group *vs.* LPS group (*n* = 3 independent experiments).

**Figure 6:**
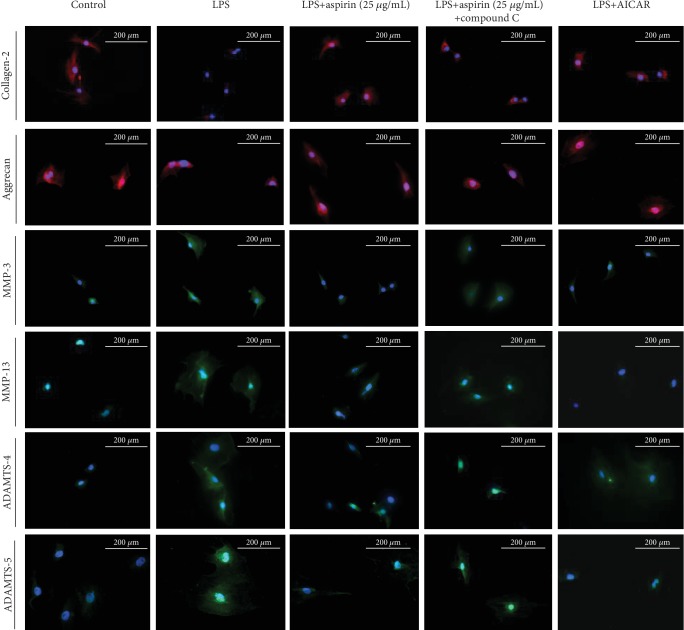
ICC staining after application of AMPK pathway agonist and inhibitor. NPCs were pretreated with an AMPK inhibitor (compound C, 100 *μ*M) or an AMPK agonist (AICAR, 500 *μ*M) for 24 h; aspirin (25 *μ*g/mL) or LPS (1 *μ*g/mL) was then added for 24 h. Subsequently, we obtained ICC results for (a) representative ICC image with COL2, aggrecan, MMP-3, MMP-13, ADAMTS-4, and ADAMTS-5 staining in NPCs. Scale bar, 200 *μ*m.

After treatment with LPS, the p-AMPK/APMK ratio was totally reversed (*P* < 0.05). Compound C, which is a specific inhibitor of AMPK, significantly decreased p-AMPK and p-ACC (Supp. Figs. [Supplementary-material supplementary-material-1]). The effect of aspirin-activated AMPK signaling pathway was blocked after pretreatment with compound C (*P* < 0.05; Figures 3(e)–3(g)). When we pretreated NPCs with compound C and then added aspirin to each group, the inhibition of ROS production was reversed (*P* < 0.05; Figures 4(a) and 4(b)). Western blotting showed that compound C increased the protein expression levels of iNOS and COX-2 but inhibited the expression of Nrf-2 (*P* < 0.05; Figures 4(c)–4(f)). Treatment with compound C and aspirin did not inhibit inflammatory cytokine expression (Figures 4(g)–4(j)). RT-PCR analysis indicated that compound C increased the expression of MMP-3, MMP-13, ADAMTS-4, and ADAMTS-5 (*P* < 0.05; Figures 5(c)–5(f)), whereas it decreased the expression of COL2 and aggrecan (*P* < 0.05; Figures 5(a) and 5(b)). ICC staining showed that COL2 and aggrecan expression decreased in NPCs, whereas that of MMP-3, MMP-13, ADAMTS-4, and ADAMTS-5 increased (Figure 6(a)). AICAR, which activates AMPK, reversed the ratio of p-AMPK to AMPK after treatment with LPS and without aspirin (*P* < 0.05; Figures 3(e)–3(g)). AICAR plays a protective role against antioxidative stress by activating the AMPK signaling pathway. Taken together, these findings indicate that aspirin attenuates LPS-induced oxidative stress and ameliorates NPC dysfunction by activating the AMPK pathway.

### 3.4. Aspirin Ameliorates IDD in the *In Vivo* Rat Model

**Figure 7:**
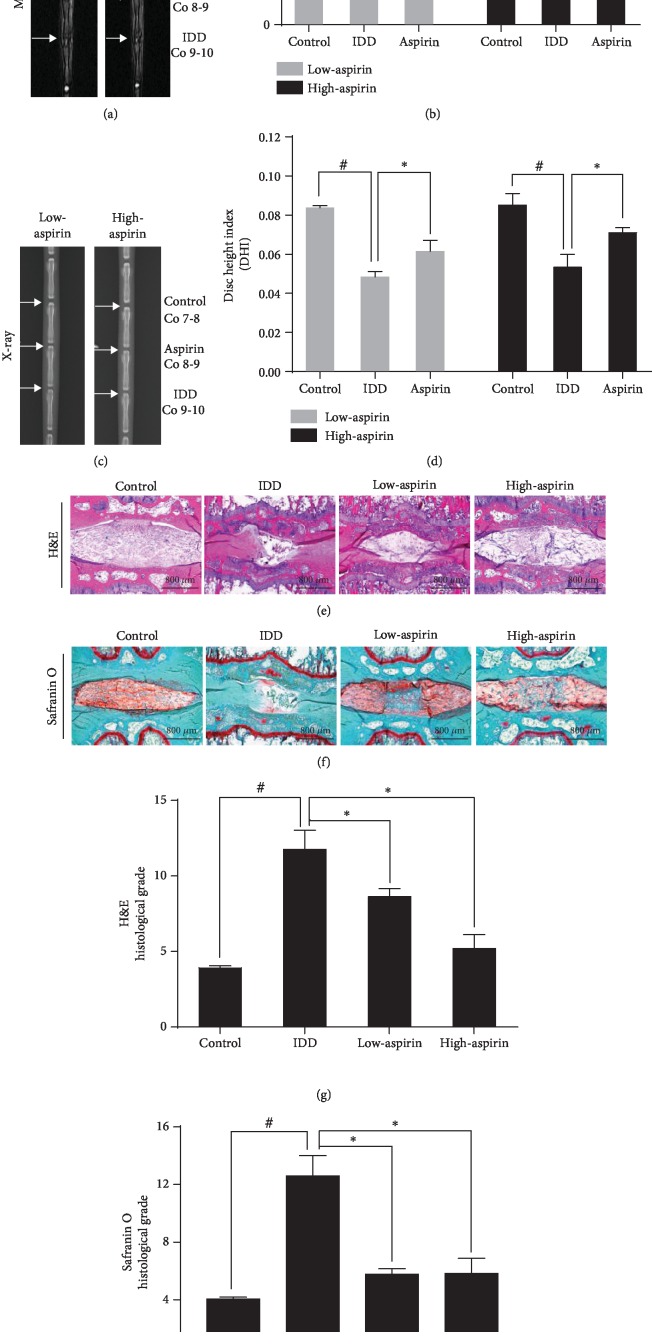
Aspirin alleviates IDD *in vivo*. SD rats' coccygeal vertebrae Co7–Co8 discs as the control group and Co8–Co10 were punctured, while those with punctured Co8–Co9 received aspirin (low dose: *n* = 10; high dose: *n* = 10) as aspirin group and punctured Co9–Co10 received PBS as the IDD group. (a) Representative MRI shows IVD signal intensity. (b) Quantitative statistics for IVD optical density (OD) value (^#^*P* < 0.05*vs*. control group; ^∗^*P* < 0.05*vs*. IDD group). (c) Representative X-ray image. (d) DHI (^#^*P* < 0.05*vs*. control group; ^∗^*P* < 0.05 vs. IDD group). Representative and histological scores of (e) H&E and (f) Safranin O Fast Green. (g) Quantitative statistics for H&E and (h) Safranin O Fast Green (^#^*P* < 0.05*vs*. control group; ^∗^*P* < 0.05*vs*. IDD group). Scale bar, 800 *μ*m. ^#^*P* and ^∗^*P* < 0.05 by one-way ANOVA and Tukey's HSD test were further analyzed between IDD group *vs*. control group, low-aspirin group vs. IDD group, high-aspirin group *vs.* IDD group (*n* = 10 independent experiments).

We successfully established an *in vivo* IDD model using SD rats (*n* = 20), then measured their degree of IDD by MRI (*n* = 10) and X-ray (*n* = 10). The MRI images obtained at the second week after puncture in the aspirin group had stronger T2-weighted signal intensities than the IDD group and ranged from ~1.26-fold to ~1.33-fold (*P* < 0.05; Figures 7(a) and 7(b)). The change in IVD height was expressed by the DHI. We observed a significant improvement in the aspirin group compared to the IDD group, which ranged from ~1.22-fold to ~1.54-fold (*P* < 0.05; Figures 7(c) and 7(d)). During the experiment, the animals remained in good condition and did not die.

After H&E (Figure 7(e)) and Safranin O Fast Green (Figure 7(f)) staining, the nucleus pulposus of the control group had many notochordal cells (vacuole-like cells) and NPCs (chondrocyte-like cells). In the IDD group, the demonstrated significantly degenerative changes were observed after puncture. The masses of notochordal cells in the nucleus pulposus were lost, and the nucleus pulposus tissue was subsequently occupied by disorganized hypocellular fibrocartilaginous tissue. Treatment with aspirin, especially high-dose aspirin, significantly inhibited the loss of nucleus pulposus tissue and alleviated the destruction of structures in IVDs compared with the IDD group (Figures 7(g) and 7(h)).

**Figure 8:**
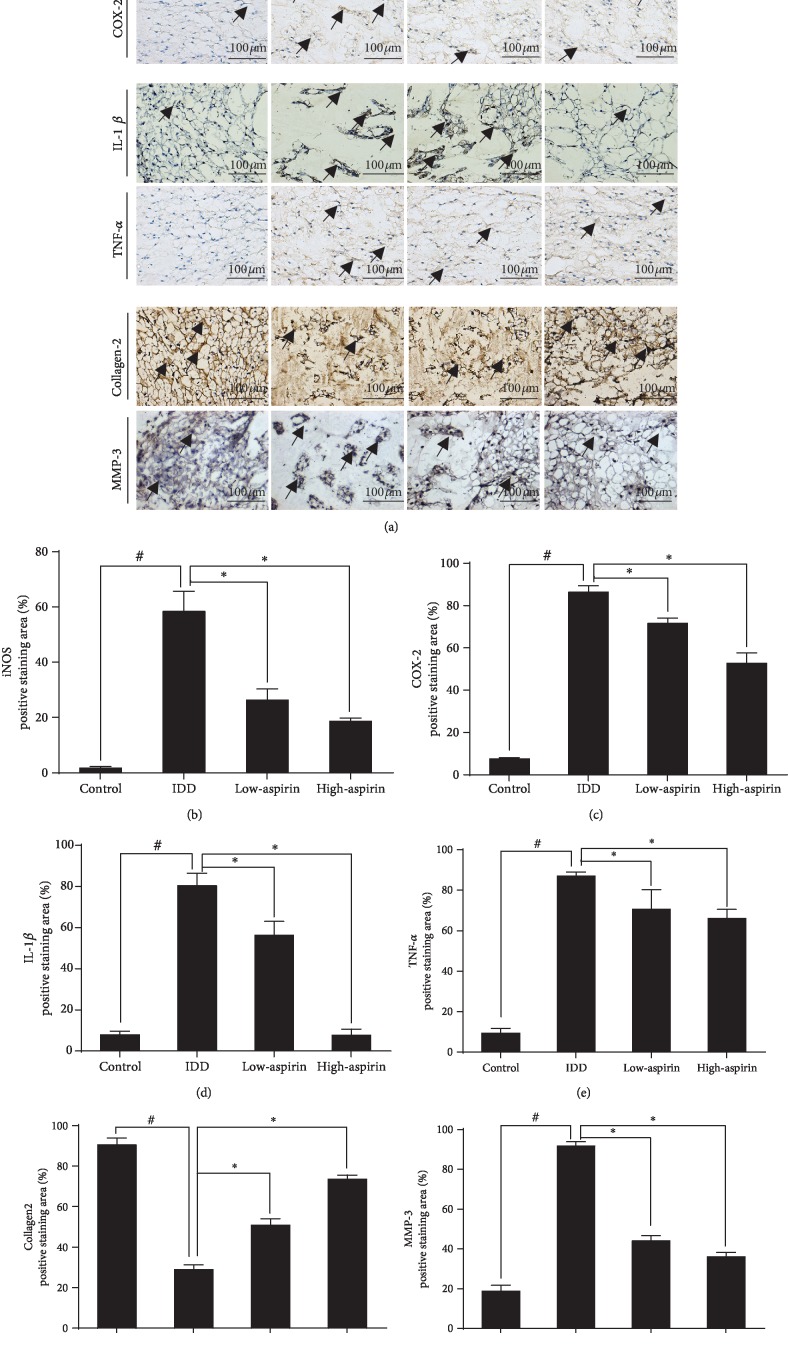
IHC staining *in vivo*. (a) Representative specimens of paraffin section with IHC staining of iNOS, COX-2, IL-1*β*, TNF-*α*, COL2, and MMP-3 in nucleus pulposus tissue; scale bar, 100 *μ*m. (b–g) The quantitative statistics for iNOS, COX-2, IL-1*β*, TNF-*α*, COL2, and MMP-3 staining (^#^*P* < 0.05*vs*. control group; ^∗^*P* < 0.05*vs*. IDD group). ^#^*P* and ^∗^*P* < 0.05 by one-way ANOVA and Tukey's HSD test were further analyzed between IDD group *vs.* control group, low-aspirin group *vs*. IDD group, and high-aspirin group *vs*. IDD group (*n* = 10 independent experiments).

By IHC staining (Figure 8(a)), we measured the expression of iNOS and COX-2 (Figures 8(b) and 8(c)), which are associated with oxidative stress, and inflammatory cytokines, including IL-1*β* and TNF-*α* (Figures 8(d) and 8(e)). All of these were attenuated by aspirin treatment. Notably, iNOS was slightly expressed in the control group but expression levels dramatically increased in the IDD group. Meanwhile, aspirin protected COL2 synthesis and prevented MMP-3 expression compared to the IDD group (Figures 8(f) and 8(g)).

## 4. Discussion

In the present study, we investigated whether aspirin could attenuate degeneration of NPCs due to LPS-induced oxidative stress and inflammation. The results showed that the protective mechanism of aspirin involves the activation of the AMPK signaling pathway that attenuates oxidative stress.

Aspirin has been widely used in the clinic as a nonsteroidal anti-inflammatory drug (NSAID) [21]. It has a long history of availability, and its lower cost and increased safety also make it the first-line treatment by numerous physicians. Usually, aspirin is taken to relieve inflammation or mild to moderate pain or fever. It inhibits the aggregation of platelets and reduces the occurrence rate of coronary-artery thrombosis or myocardial infarction [35]. Some epidemiological studies have suggested that long-term use of low-dose aspirin might prevent cancer [36]. Numerous studies have revealed that aspirin may have more potential applications. Piazza et al. found that NSAIDs could induce cell apoptosis and alter the expression of cell cycle regulatory genes [37, 38]. Redlak et al. [39] reported that aspirin could inhibit the activity of protein kinase C, an upstream regulator of activation of reduced *β*-nicotinamide adenine dinucleotide 2′-phosphate (NADPH) oxidases. In this study, we found that aspirin could activate AMPK to attenuate oxidative stress and inhibit NPC dysfunction. These findings indicate that aspirin might be considered as a novel option for mitigating IDD.

Oxidative stress is an important factor in the progression of IDD [12, 13]. Studies have shown that factors, such as aging or cellular senescence, malnutrition, and some diseases, such as type II diabetes, could induce oxidative stress in IVDs [40–42]. Notably, NPCs are in hypoxic environments and gain energy mainly through glycolysis [16]. These produce ROS via oxidative metabolism, but overproduction of ROS directly induces damage to NPCs and perturbs the homeostasis of the disc matrix. For example, ROS may reduce synthesis of proteoglycans and increase MMP expression [43]. Moreover, many proinflammatory cytokines can significantly stimulate production of ROS, especially in degenerated or aged IVDs [16]. In addition, NO is an important inflammatory modulator in osteoarthritic cartilage. Earlier studies have shown that reducing NO production by inhibiting iNOS can significantly reduce production of important catabolic factors, such as metalloproteinases and IL-1*β* [44]. In our study, aspirin significantly decreased iNOS and NO, which indicates that it attenuates oxidative stress in IDD.

AMPK is a serine/threonine protein kinase that is uniquely expressed in mammalian cells. It participates in many biological functions, such as regulating energy balance and cell metabolism [45]. The AMPK signaling pathway has been extensively studied in metabolic diseases, such as diabetes. Some researchers have found that AMPK mediates energy metabolism at the intracellular level and can suppress various pathological oxidative stressors or inflammatory responses [46]. Our results showed that aspirin could activate the AMPK signaling pathway in NPCs. Recently, Hawley et al. [47] found that salicylate inhibits dephosphorylation of the activating phosphorylation site threonine-172 and activates the AMPK signaling pathway, which agrees with our results. In addition, we found that preinhibition of the AMPK pathway reversed the protective effects of aspirin on inflammation, oxidation, and thereby NPC dysfunction, which further demonstrates that aspirin attenuates IVD by activating the AMPK signaling pathway. To our knowledge, this is the first study that has shown that aspirin can attenuate oxidative stress in NPCs through this pathway. Hawley et al. [47] also found that salicylate activates AMPK and bound to the same site at which a synthetic activator (A-769662) induces allosteric activation of AMPK. We have shown that the activity of the AMPK signaling pathway leads to a significant decrease in the downstream production of oxidative stressors, including ROS and iNOS. Meanwhile, through the inhibition of the AMPK signaling pathway, compound C promotes oxidative stress [48] and increases ROS expression [49].

We observed an interesting and notable phenomenon in our current study: increased iNOS led to an increase in downstream output of NO, and activating AMPK attenuated the expression of iNOS. This finding is the same as those of Tsoyi et al. [50] and Chen et al. [51] in NPCs but is in contrast with the results of Lee et al. [52], who found that AMPK promotes the expression of iNOS and production of NO in kidney proximal tubular epithelial cells. Their results seemed to show that iNOS is a positive factor that protects cells from an oxidative stress environment. However, iNOS inhibition did not slow the progression of osteoarthritis in human patients. In the current study, we observed that the application of a high dose of aspirin significantly attenuated the production of iNOS, NO, and ROS and subsequently suppressed NPC dysfunction *in vitro* and reverses the IDD process *in vivo*. We hypothesize that the pathogenic mechanisms of osteoarthritis might be different from those of IDD and iNOS might play different physiological roles in various cell lines.

## 5. Conclusions

Here, we demonstrated that aspirin activates the AMPK signaling pathway to ameliorate the process of IVD deterioration. Oxidative stress is an important pathological change in IDD, and effectively relieving it could potentially be a novel target for the treatment of IDD.

## Data Availability

The data used to support the findings of this study are available from the corresponding authors upon request.

## Supplementary Materials

Supplementary Materials RT-PCR detection of the applied primer sequences. Primer sequence design by querying the NCBI database. Supplementary Figure 1: LPS induced oxidative stress and degeneration in NPCs. (A) We cultured NPCs in different concentrations of LPS (0, 0.01, 0.1, 1, and 10 μg/mL) for 1, 3, and 5 days and then detected cell viability by CCK-8 (^∗^*P* < 0.05 vs. control group). (B) Fluorescence images showed ROS levels in different groups. Scale bar, 400 *μ*m. (C, D) Average fluorescence intensity of ROS was detected by FCM (^∗^*P* < 0.05 vs. control group). (C) The NPCs were cultured in different concentrations of LPS (0, 0.01, 0.1, and 1 μg/mL) for 24 hours. (D) The NPCs were cultured with LPS (1 μg/mL) in different hours (0, 0.25, 0.5, 1, 3, 6, 12, and 24 hours). NPCs were pretreated with LPS (1 *μ*g/mL) for 12 h. Subsequently, we observed ICC staining of COL2 and MMP-3 in NPCs. (E) Representative image of ICC staining of COL2 and MMP-3 in NPCs. Scale bar, 200 *μ*m. (F) Western blot results of iNOS, COX-2, and Nrf-2 and (G–I) the quantitative statistic, respectively (^∗^*P* < 0.05 vs. control group). (J–M) RT-PCR results of COL2, aggrecan, iNOS, and COX-2 (^∗^*P* < 0.05 vs. control group; N.S. = no statistical significance). ^∗^*P* < 0.05 by one-way ANOVA and Tukey's HSD tests was further analyzed between each group vs. control (*n* = 3 independent experiments). Supplementary Figure 2: the effect of compound C or AICAR on the AMPK signaling pathway. NPCs were treated solely with an AMPK inhibitor (compound C, 100 *μ*M) or an AMPK agonist (AICAR, 500 *μ*M) for 24 h. (A, B) Western blot results of p-AMPK, AMPK, p-ACC, and ACC. (C, D) Densitometric analysis of p-AMPK/AMPK and p-ACC/ACC levels, respectively (^∗^*P* < 0.05 vs. control group). ^∗^*P* < 0.05 by one-way ANOVA and Tukey's HSD tests was further analyzed between AICAR group and compound C group vs. control group (*n* = 3 independent experiments).
