# Supplementary material for: Aspirin-Mediated Attenuation of Intervertebral Disc Degeneration by Ameliorating Reactive Oxygen Species In Vivo and In Vitro
Source: Oxid Med Cell Longev. 2019 Nov 6;2019:7189854. doi: 10.1155/2019/7189854 (PMC6874877; doi:10.1155/2019/7189854)
Supplement: Supplementary Materials — RT-PCR detection of the applied primer sequences. Primer sequence design by querying the NCBI database. Supplementary Figure 1: LPS induced oxidative stress and degeneration in NPCs. (A) We cultured NPCs in different concentrations of LPS (0, 0.01, 0.1, 1, and 10 μg/mL) for 1, 3, and 5 days and then detected cell viability by CCK-8 (∗P < 0.05 vs. control group). (B) Fluorescence images showed ROS levels in different groups. Scale bar, 400 μm. (C, D) Average fluorescence intensity of ROS was detected by FCM (∗P < 0.05 vs. control group). (C) The NPCs were cultured in different concentrations of LPS (0, 0.01, 0.1, and 1 μg/mL) for 24 hours. (D) The NPCs were cultured with LPS (1 μg/mL) in different hours (0, 0.25, 0.5, 1, 3, 6, 12, and 24 hours). NPCs were pretreated with LPS (1 μg/mL) for 12 h. Subsequently, we observed ICC staining of COL2 and MMP-3 in NPCs. (E) Representative image of ICC staining of COL2 and MMP-3 in NPCs. Scale bar, 200 μm. (F) Western blot results of iNOS, COX-2, and Nrf-2 and (G–I) the quantitative statistic, respectively (∗P < 0.05 vs. control group). (J–M) RT-PCR results of COL2, aggrecan, iNOS, and COX-2 (∗P < 0.05 vs. control group; N.S. = no statistical significance). ∗P < 0.05 by one-way ANOVA and Tukey's HSD tests was further analyzed between each group vs. control (n = 3 independent experiments). Supplementary Figure 2: the effect of compound C or AICAR on the AMPK signaling pathway. NPCs were treated solely with an AMPK inhibitor (compound C, 100 μM) or an AMPK agonist (AICAR, 500 μM) for 24 h. (A, B) Western blot results of p-AMPK, AMPK, p-ACC, and ACC. (C, D) Densitometric analysis of p-AMPK/AMPK and p-ACC/ACC levels, respectively (∗P < 0.05 vs. control group). ∗P < 0.05 by one-way ANOVA and Tukey's HSD tests was further analyzed between AICAR group and compound C group vs. control group (n = 3 independent experiments). [file 7189854.f1.docx]

**Supplementary Table**

**Table 1. RT-PCR primer sequences**

| Gene name | NCBI ID | Forward primer (5’-3’) | Reverse primer (5’-3’) |
| --- | --- | --- | --- |
| GAPDH | 24383 | GCAAGTTCAACGGCACAG | CGCCAGTAGACTCCACGAC |
| Collagen 2 | 25412 | GAGTGGAAGAGCGGAGACTACTG | CTCCATGTTGCAGAAGACTTTCA |
| Aggrecan | 58968 | TACGACGCCATCTGCTACAC | TCGAAGATGGGCTTTGCAGT |
| MMP-3 | 171045 | TTTGGCCGTCTCTTCCATCC | GCATCGATCTTCTGGACGGT |
| MMP-13 | 171052 | TCCATCCCGAGACCTCATGT | AGCATCATCATAACTCCACACG |
| ADAMTS-4 | 66015 | CGTTCCGCTCCTGTAACACT | TTGAAGAGGTCGGTTCGGTG |
| ADAMTS-5 | 304135 | GCCTGCAAGGGAAATGTGTG | GGCGGAAAGATTTGCCGTTAG |
| COX-2 | 29527 | GATGACGAGCGACTGTTCCA | TGGTAACCGCTCAGGTGTTG |
| iNOS | 24599 | TCCTCAGGCTTGGGTCTTGT | AGAAACTTCCAGGGGCAAGC |

**Supplementary Figure and Figure Legend**

**Figure 1**


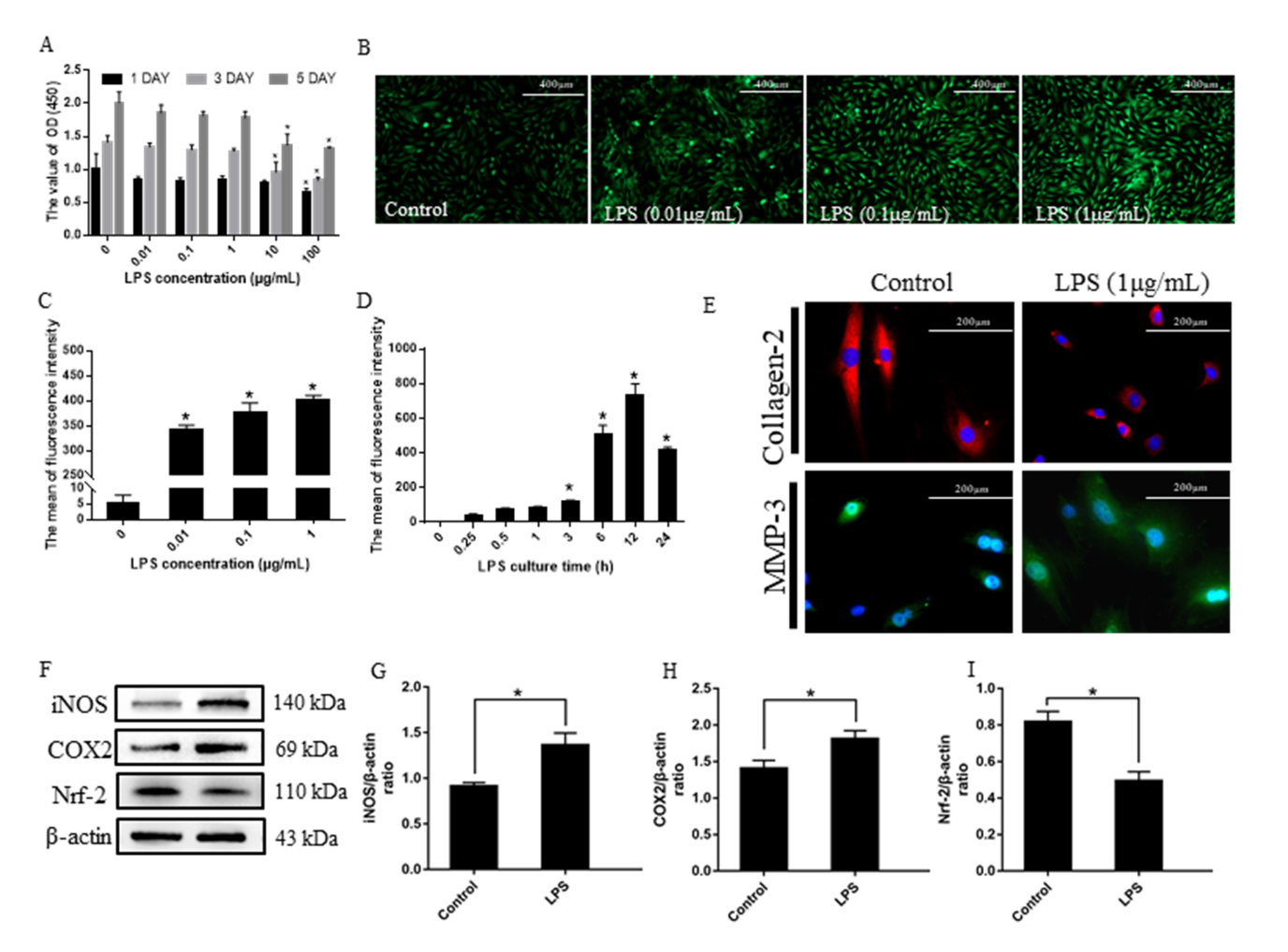


**Supplementary Figure 1. LPS induced oxidative stress and degeneration in NPCs.** **(A)** We treated NPCs with different concentrations of LPS (0, 0.01, 0.1, 1, and 10 μg/mL) for 1, 3, and 5 days and then detected cell viability by CCK-8 (**P* < 0.05 vs. control group). **(B)** Fluorescence images showed ROS levels in different groups. Scale bar, 400 μm. **(C–D)** Average fluorescence intensity of ROS was detected by FCM (**P* < 0.05 vs. control group). **(C)** NPCs were treated with different concentrations of LPS (0, 0.01, 0.1, and 1 μg/mL) for 24 hours. **(D)** NPCs were treated with 1 μg/mL LPS for different time periods (0, 0.25, 0.5, 1, 3, 6, 12, 24 hours). **(E)** NPCs were pretreated with LPS (1 μg/mL) for 12 h. Subsequently,

we observed ICC staining of COL2 and MMP-3 in NPCs. Representative images of ICC staining of COL2 and MMP-3 in NPCs. Scale bar, 200 μm. **(F)** Western blot results of iNOS, COX-2, and Nrf-2 and **(G-I)** densitometric analysis of protein levels, respectively (**P* < 0.05 vs. control group). **P* < 0.05 by one-way ANOVA and Tukey’s HSD tests was further analyzed between LPS group vs. control group. (n = 3 independent experiments).

**Figure 2**

**
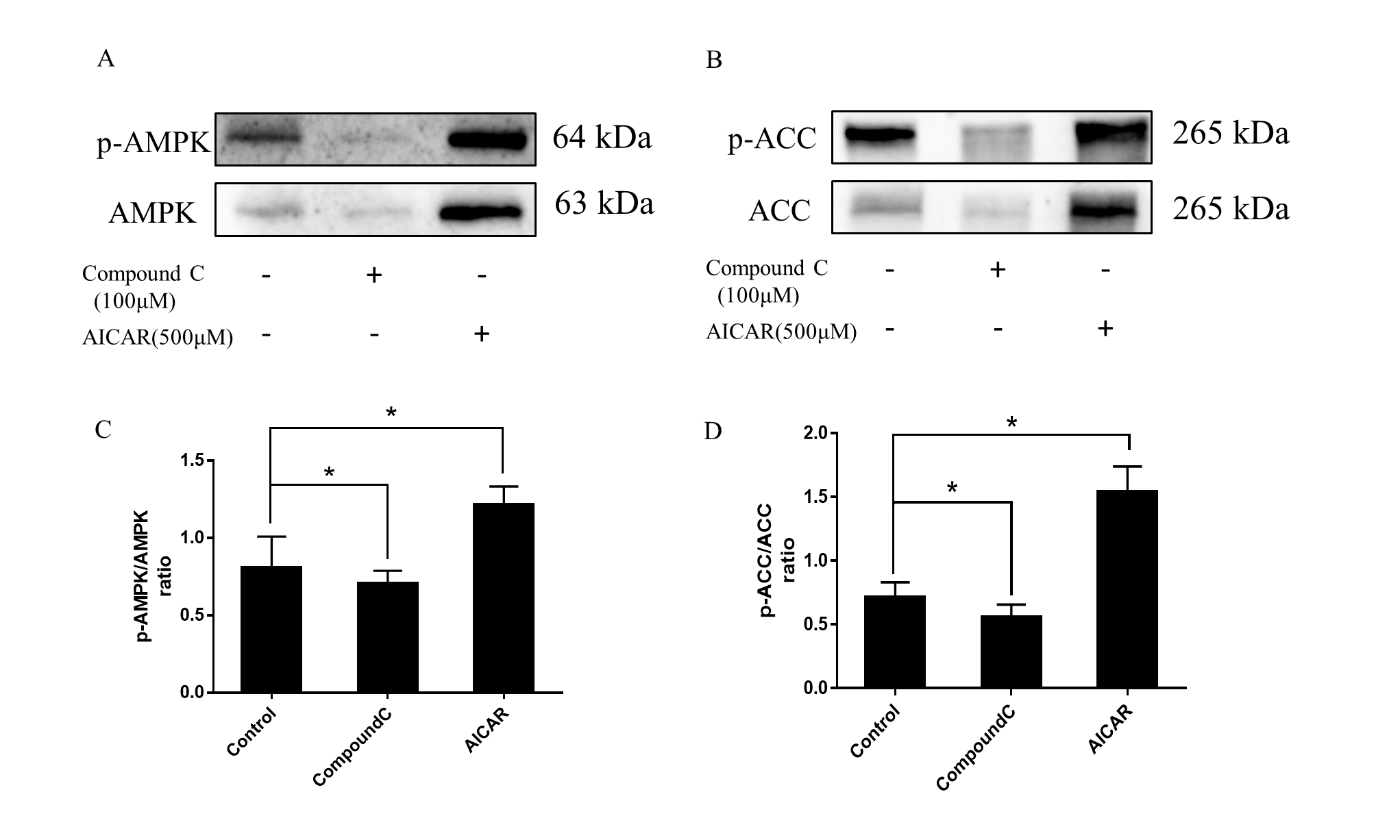
**

**Supplementary Figure 2. The effect of Compound C or AICAR on the AMPK signaling pathway.** NPCs were treated solely with an AMPK inhibitor (compound C, 100 μM) or an AMPK agonist (AICAR, 500 μM) for 24 h. **(A-B)** Western blot results of p-AMPK, AMPK, p-ACC and ACC. **(C-D)** Densitometric analysis of p-AMPK/AMPK and p-ACC/ACC levels, respectively (**P* < 0.05 vs. control group). **P* < 0.05 by one-way ANOVA and Tukey’s HSD tests was further analyzed between AICAR group or Compound C group vs. control group. (n = 3 independent experiments).
